# Digital Mapping of Soil Organic Carbon Contents and Stocks in Denmark

**DOI:** 10.1371/journal.pone.0105519

**Published:** 2014-08-19

**Authors:** Kabindra Adhikari, Alfred E. Hartemink, Budiman Minasny, Rania Bou Kheir, Mette B. Greve, Mogens H. Greve

**Affiliations:** 1 Department of Soil Science, University of Wisconsin−Madison, Madison, Wisconsin, United States of America; 2 Department of Environmental Sciences, The University of Sydney, Sydney, New South Wales, Australia; 3 Department of Agro-ecology, Aarhus University, Tjele, Denmark; Tennessee State University, United States of America

## Abstract

Estimation of carbon contents and stocks are important for carbon sequestration, greenhouse gas emissions and national carbon balance inventories. For Denmark, we modeled the vertical distribution of soil organic carbon (SOC) and bulk density, and mapped its spatial distribution at five standard soil depth intervals (0−5, 5−15, 15−30, 30−60 and 60−100 cm) using 18 environmental variables as predictors. SOC distribution was influenced by precipitation, land use, soil type, wetland, elevation, wetness index, and multi-resolution index of valley bottom flatness. The highest average SOC content of 20 g kg^−1^ was reported for 0−5 cm soil, whereas there was on average 2.2 g SOC kg^−1^ at 60−100 cm depth. For SOC and bulk density prediction precision decreased with soil depth, and a standard error of 2.8 g kg^−1^ was found at 60−100 cm soil depth. Average SOC stock for 0−30 cm was 72 t ha^−1^ and in the top 1 m there was 120 t SOC ha^−1^. In total, the soils stored approximately 570 Tg C within the top 1 m. The soils under agriculture had the highest amount of carbon (444 Tg) followed by forest and semi-natural vegetation that contributed 11% of the total SOC stock. More than 60% of the total SOC stock was present in Podzols and Luvisols. Compared to previous estimates, our approach is more reliable as we adopted a robust quantification technique and mapped the spatial distribution of SOC stock and prediction uncertainty. The estimation was validated using common statistical indices and the data and high-resolution maps could be used for future soil carbon assessment and inventories.

## Introduction

Digital Soil Mapping uses statistical tools to quantify the spatial relationship between soil property values to its environmental covariates [1]. The digital mapping of soil organic carbon (SOC) at fine resolution is a challenging task [2] and the mapping is also a high priority for SOC assessment and monitoring [3]. Spatial models on SOC prediction has a long history (e.g., [4]). A range of techniques have been used to predict and map SOC from landscape to national or continental levels and Minasny et al. [5] provided a comprehensive review. Several researchers applied splines to model the vertical distribution of SOC in the soil profiles and predicted SOC at a landscape scale using data-mining tools and environmental variables as predictors [6]–[8]. Mishra et al. [9] calculated SOC pool in each soil horizon and applied geographic weighted regression to map SOC pool at a regional scale in mid-west USA. Odgers et al. [10] used splines to derive SOC content from soil map units and predicted SOC at six standard soil depths for the entire USA. Arrouays et al. [11], Bou Kheir et al. [12], Chaplot et al. [13], Doblas-Miranda et al. [14], mapped SOC at national level using different statistical tools ranging from statistical aggregation to advanced regression tools such as regression trees.

The distribution of SOC changes across the landscape and it also varies by depth. In most soils, SOC is higher in the surface horizons and it decreases with depth. Such depth-wise variability is mostly continuous [15]–[17] except in soils with a strong human impact like some soils in the Netherlands [18]. Although most SOC studies and inventories are confined to 30 cm soil depth [19]–[21], the amount of SOC stored below 30 cm is of relevant in many ecosystems [22], [23]. For accurate quantification of SOC stocks, a depth function needs to be modeled. Several tools exist: spline function [15], [16], exponential decay function [24] or soil-type specific or profile depth functions [18], [25]. For modeling SOC with depth, the equal-area spline function has been proven to be useful in several studies [7], [8], [10]. Spline predicted SOC values with depth act as a geo-referenced point data to which environmental variables are joined and prediction models are generated using digital soil mapping techniques.

In Denmark, studies on SOC dynamics and its quantification has started after a national wide soil database was established between the years 1975–1985. Based on simple statistical scaling-up techniques, Krogh et al. [26] calculated a total stock of about 579 Tg and reported that 69% of it was stored in the soils under agriculture. Greve et al. [27] estimated topsoil SOC contents (g 100 g^−1^) for the whole country but have not assessed the SOC stocks. Bou Kheir et al. [12] predicted the spatial extent of organic soils across Denmark. Similarly, Olesen [28] and Taghizadeh-Toosi et al. [29] estimated the stock but did not map its distribution. Most of these studies have estimated the SOC content and stock but they have not explored the spatial distribution of SOC stock nor quantified the uncertainty of the SOC predictions.

We applied digital soil mapping techniques to quantify the SOC content and stocks for Denmark. The main objectives of this study were: to model the vertical distribution of SOC content and bulk density in soil profiles, to predict and map their spatial distribution using environmental variables, to identify major environmental variables responsible for SOC distribution, to estimate the SOC stock for the soils of Denmark, and to assess the uncertainties in the SOC predictions.

## Materials and Methods

### Study area

Denmark is situated in Northern Europe covering an area of approximately 43,000 km^2^. The temperate climate is characterized by a mild winters with annual mean winter and summer temperatures of about 0°C and 16°C [30]. Precipitation is well distributed throughout the year with an average annual rainfall of 800 mm in the west to 500 mm to the east. The country is relatively flat with a mean elevation above mean sea level is about 31 m. Denmark is divided into 10 physiographic regions−referred in this paper as geo-regions−based on geographical, climatic and soil-formation criteria.

The soils are coarse sandy to clayey to heavy clays as defined in the Danish Soil Classification System [31]. Soil in the western part of the country are developed in non-glaciated sandy parent material along the glacial flood-plains and Saalian moraine, whereas the soils from the central and eastern region have been developed on relatively young basal till high in finer materials [31]. Most of the soils in the north have been formed in sand mixed with uplifted marine sediments. More than 66% of the soils are classified as Podzols (Spodosols) and Luvisols (Alfisols). Podzols occupy a major portion in the west and Luvisols and Cambisols (Inceptisols, Entisols) in the central and eastern part of the country [32]. Peat soils (Histosols) occur in poorly drained basins throughout the country. About 66% of the total land area is used for agriculture with grain and potato as the main crops. Forest areas include spruce, pine and beach and these cover more than 12%.

### Point data

Point (pedon) data on soil organic carbon (SOC) (g kg^−1^) and bulk density (*D_b_*) (Mg m^−3^) were derived from two databases: Danish Soil Classification database (DSC) and Danish Soil Profile database (DSP). DSC consists of about 36,000 point observations from the topsoil (0−20 cm) sampled randomly from agricultural fields in the period 1975−1980. From about 6,000 same pedons, soils from the subsoil (35−55 cm) was also sampled. SOC content was determined by dry combustion using a LECO IR-12 furnace. DSP consists of a grid based data (7×7 km spacing) established during the 1990s for improve fertilizer recommendation in Denmark [33]. At each 850 grid intersection, soil samples were collected based on genetic horizons and were analyzed for SOC by dry combustion. For some selected horizons, samples were taken to determine *D_b_*. In addition, soil data from about 1100 profiles were used, and these were collected during the establishment of main gas pipeline system and other research activities across Denmark [34].

In total 40,250 topsoil point samples and 1,994 soil profiles with horizon based SOC data were used in this study. About 1,133 soil profiles included *D_b_* measurements.

### Environmental covariates

The environmental covariates data used in this study are terrain parameters from the Digital Elevation Model (DEM) of Denmark derived using Light Detection and Ranging (LiDAR) technology. The LiDAR points were interpolated using Delaunay Triangulation and the output Triangular Irregular Network (TIN) surface was converted to a grid DEM with a 1.6×1.6 m spacing. This study resampled the original 1.6 m DEM to 30.4 m using simple aggregation considering the mean value. This 30.4 m grid size was also used in the previous studies in Denmark (e.g., [32], [35]). Before aggregating to a coarser grid, the DEM was corrected by removing the pits and peaks of about 50 cm dimensions in order to ensure a regular flow on the surface. Once the DEM was processed, a number of terrain parameters (e.g., slope aspect, slope gradient, elevation, SAGA wetness index, multi-resolution index of valley bottom flatness (MrVBF), altitude above channel network, slope-length factor, over-land flow distance, and valley depth) were derived. Tools and algorithms incorporated in SAGA GIS [36] and Arc GIS V10.2 [37] were used to process the DEM and to derive these parameters.

Other covariates used were six choropleth maps which were compiled at different cartographic scales including: soil map, landscape types, geo-regions, geology, land use, and wetlands – see Table 1 and for more detail Adhikari et al. [35].

**Table 1:** List of environmental variables used to predict the distribution of soil organic carbon and its stock in Denmark.

Environmentalvariables	Scorpanfactor	Type ofvariable	Description	Range ofvalues	Scale orresolution	Reference
Soil map	S	Categorical	Map of Soil types based onsoil texture (8 classes)	-	1∶50,000	[31]
Precipitation	C	Continuous	Average annual rainfall(mm) (1961−1990)	525 to 905	30.4 m	[31]
Geo-regions	C	Categorical	Scanned geographicalregions map (10 classes)	-	1∶100,000	[31]
Insolation	C	Continuous	Potential incomingsolar radiation (2011)	254 to 698	30.4 m	[61]
Mid-slope position	C, N	Continuous	Covers the warmerzones of slopes	0 to 1	30.4 m	[62]
Land use	O	Categorical	CORINE Land coverdata adopted inDenmark (31 classes)	-	1∶100,000	[45]
Elevation	R	Continuous	Elevation of the land surfacederived from LiDAR (m)	0 to 170	30.4 m	
Slope gradient	R	Continuous	Maximum rate of changebetween the cellsand neighbors (degree)	0 to 90	30.4 m	[63]
Slope aspect	R	Continuous	Direction of the steepestslope from the North (degree)	0 to 360	30.4 m	[63]
Flow accumulation	R	Continuous	Number ofupslope cells	1 to 73645	30.4 m	
SAGA wetness index	R	Continuous	Wetness Index.WI = ln (A_s_ / tan β): where A_s_ ismodified catchment areaand β is the slope gradient	7.2 to 19	30.4 m	[64]
Multi-resolutionvalley bottom flatness	R	Continuous	Possible depositional areas	0 to 11	30.4 m	[65]
Valley depth	R	Continuous	Extent of the valley depth (m)	0 to 90	30.4 m	
Wetlands	S, R	Categorical	Map showing the presenceor absence of wetlands	-	1∶20,000	[31]
Landscape	R	Categorical	Landform types (10 classes)	-	1∶100,000	[31]
Altitude abovechannel network	R	Continuous	Vertical distance tochannel network base level (m)	0 to 56	30.4 m	
Slope length factor	R	Continuous	LS-factor of UniversalSoil Loss Equation (m)	0 to 47	30.4 m	[66]
Geology	P	Categorical	Scanned and registeredgeological map (86 classes)	-	1∶100,000	[31]

*S-soil types; C-climate, O-organisms; R-relief; P-parent material; N-spatial position.*

**Figure 1:**
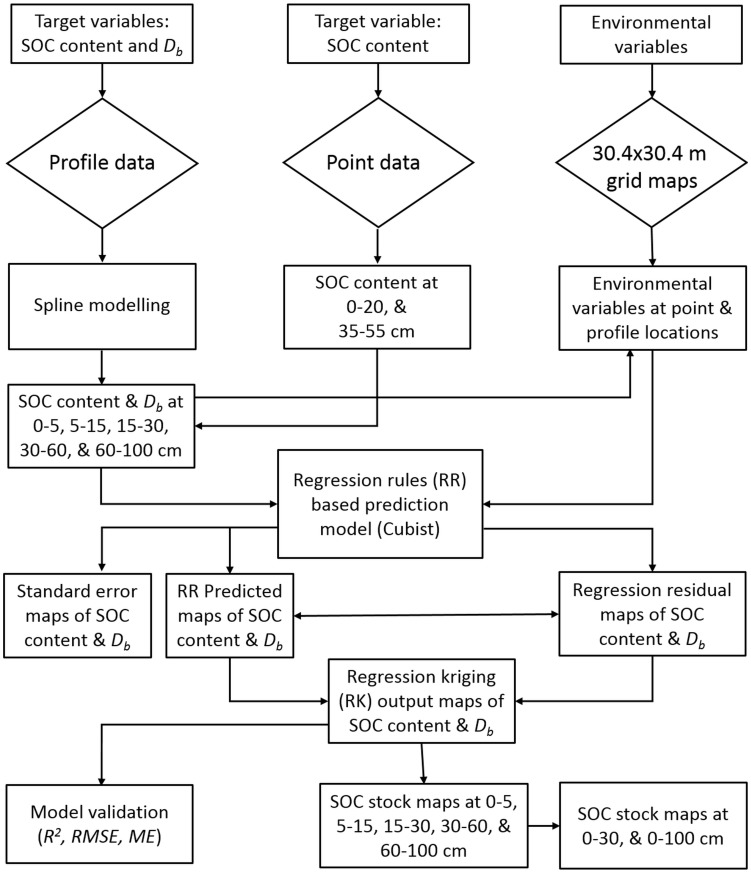
Schematic representation of overall prediction scenario.

Key environmental variables affecting the spatial distribution of SOC and *D_b_* in Denmark were identified. The relative usage of the environmental variables used during SOC and *D_b_* prediction was calculated for each depth and their importance was expressed in percentage. This was done with *Cubist* software which determines the relative importance of variables based on their usage in the prediction rules. The prediction method adopted in this study is summarized in Figure 1.

### Modeling SOC and D_b_ distribution

The vertical distribution of SOC and *D_b_* in the soil profiles was modeled with mass preserving equal-area quadratic splines [15] in R [38]. The mathematical derivation of the spline has been described by Malone et al. [8]. As the fitting quality of splines to profile attribute data depends on a smoothing parameter−lambda (λ), we tested seven λ values (0.00001, 0.0001, 0.001, 0.01, 0.1, 1, 10) for SOC and *D_b_* data from all the profiles and selected a λ value that showed the best fit for all the profiles using the root mean square. With increasing λ value, the fit becomes more rough. During the fit, we pegged the spline by introducing a 1 cm thick slice with a same SOC value at the topmost layer to prevent unnecessary extrapolation on the surface horizon.

Once the depth function of SOC and *D_b_* were modeled, a weighted-average value of these properties were derived for five soil depths (0−5, 5−15, 15−30, 30−60, and 60−100 cm) based on the GlobalSoilMap specifications [39]. To these new values for the standard depths from all soil profiles, environmental variables were intersected and used for statistical analysis and spatial prediction.

### Mapping to the spatial domain

Spatial prediction of SOC content and *D_b_* at five depths was based on Regression kriging (RK) [40]. RK assumed that the spatial prediction function consists of a deterministic model formed by a regression, and the residuals of the regression (unexplained variation) are spatially correlated. The general principle of RK includes (1) regression, and (2) simple kriging of the residuals from the regression, where outputs from these two steps are added to obtain the final prediction. For the regression step, we adopted Regression-rules (RR) derived using *Cubist* software [41]. This tool generates a set of condition−based rules where each rule comes with a multiple regression prediction function that only operates once the conditions specified by the rule are met [42].

To build the SOC and *D_b_* prediction model in *Cubist*, the data set was split randomly into two sets: 75% for calibration and 25% for model validation. Before the data split, SOC content from the topsoil observations (0−20 cm) were joined to the spline predicted SOC content from 0−5 and 5−15 cm soil depths. Similarly, SOC content from subsoil observations (35−55 cm) were attached to the spline predicted SOC at 30−60 cm soil depth. This way, a larger number of point SOC observations were incorporated to the splined SOC data from the 7×7 km grid profiles. The *Cubist* tool was run for log transformed SOC [log SOC g kg^−1^] and *D_b_* data from each depth interval and the output was converted to a regular grid map using a program written in FORTRAN. For each calibration location, the difference between measured and RR predicted value was calculated and its spatial distribution over the study area was generated using local variogram and point kriging in VESPER program [43]. This continuous residual surface was added to the corresponding RR output to get a final prediction of SOC and *D_b_* for all five depths. Together with the prediction, a map showing the uncertainty of the prediction was generated. Both SOC prediction and uncertainty maps at each depth were then back-transformed to SOC in g kg^−1^.

### Bulk density in the peat areas

Peat lands are mostly present along the central part of the wetlands across Denmark. Bulk density in those areas were adjusted according to Greve et al. [44]. For the three surface layers (i.e., 0−5, 5−15, and 15−30 cm soil depths), *D_b_* from 0−30 cm was used, and for the 30−60 and 60−100 cm, *D_b_* from 30−60 cm, and 60−90 cm were used (Table 2).

**Table 2:** Average soil bulk density (Mg m^−3^) for different soil organic carbon levels (g 100 g^−1^) within the central wetlands [Source: [44]].

SOC content	Soil depth (cm)		
	0−30	30−60	60−90
<6	1.15	0.56	0.76
6−12	0.77	0.61	0.44
>12	0.39	0.25	0.19

### SOC stocks

SOC stock for each soil depth was calculated according to Eq. (1) using SOC content and *D_b_* data. SOC stocks from the five layers were summed to obtain a SOC stock to 1 m soil depth. *D_b_* in Eq. (1) was corrected for gravel content.
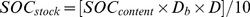
(1)Where *SOC_stock_* is the SOC stock (t ha^−1^), *SOC_content_* the SOC content (g kg^−1^), *D_b_* the soil bulk density (Mg m^−3^) and *D* the given soil layer thickness (cm).

### Model validation

Model performance in predicting SOC content and *D_b_* was evaluated on 25% of the point data. The following three indices that were calculated:
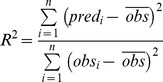
(2)

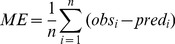
(3)

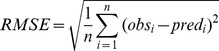
(4)Where *obs* and *pred* are observed and predicted SOC and *D_b_* values from *n* number of observations at *i* ^th^ locations, *ME* is mean error, and *RMSE* is the root mean square error.

Predicted SOC stock was determined for all five depth intervals and then aggregated to 0−30 cm and 0−100 cm soil depth. These two C stock maps were stratified based on soil and land use types. The soil class map of Denmark based on the FAO legend [32] and a land use map (CORINE data) were used. The CORINE database for Denmark has 31 classes [45] but in this study the legend was reduced to five major land uses types: artificial surface (urban areas, industrial areas, road network and ports etc.), agricultural areas, forest and semi-natural areas, wetlands, and others (e.g., coastal lagoon, estuaries etc.) for ease of comparison to other studies (e.g., [11], [22], [26]). We also stratified our stock maps based on Danish geo-regions. We first derived the area of each soil, land use and geo-regions class based on the number of predicted pixels within those classes and then calculated an average and total SOC stock for each class at 0−30 and 0−100 cm soil depth. The 0−30 cm depth represents the plough depth from agriculture areas and estimation of carbon for this depth is of interest to farm management. The top 1 m soil depth mostly represents a rooting depth of many field crops and may act as an important soil depth section for carbon balance and accounting studies.

## Results

### Summary of SOC and bulk density

SOC content was highly variable and ranged from 0 to 562 g kg^−1^ for the topsoil (0−20 cm) and from 0 to 569 g kg^−1^ in the subsoil (Table 3). Mean SOC decreased with soil depth and SOC at 60−100 cm was about four times lower than the SOC in the 0−5 cm layer. With depth, the coefficient of variation (CV) of the SOC content increased. The CV at 0−5 cm was 184% and that for the 60−100 cm was about 466%. The SOC data was positively skewed at all soil depths with a maximum skewness coefficient at 60−100 cm. The equal-area splines modeled the depth-wise distribution and generated a continuous SOC profile to 1 m depth. The best λ value to fit all soil profiles for both SOC and *D_b_* data was 0.1. Also average *D_b_* was found to be increased with soil depth. Up to 30 cm depth the *D_b_* was on average 1.44 Mg m^−3^, whereas it increased to 1.52 Mg m^−3^ below 60 cm depth. Bulk density appeared to be less variable with depth (Table 3).

**Table 3:** Descriptive statistics of soil organic carbon content (g kg^−1^) and bulk density (*D_b_*) (Mg m^−3^) data used in this study.

Parameters	Spline predicted data										Point observation		
	Soil depth (cm)												
	0−5		5−15		15−30		30−60		60−100			0−20	35−55
	SOC	*D_b_*	SOC	*D_b_*	SOC	*D_b_*	SOC	*D_b_*	SOC	*D_b_*		SOC	
Minimum	0.07	0.47	0.09	0.47	0.02	0.47	0.02	0.47	0.01	0.47		0.10	0.10
Maximum	562.31	1.84	562.1	1.84	562.22	1.99	564.01	2.01	570.01	1.96		562.21	559.22
Interquartile Range	15.92	0.21	14.11	0.20	10.22	0.19	6.12	0.17	2.33	0.16		9.16	7.61
Mean	35.22	1.44	30.71	1.44	23.81	1.46	15.61	1.52	9.91	1.59		19.71	15.01
Std. error of mean	1.45	0.00	1.18	0.00	1.11	0.00	1.14	0.00	1.03	0.00		0.07	0.48
Std. deviation	64.81	0.17	52.9	0.17	49.74	0.15	51.2	0.15	46.41	0.15		15.38	44.85
Coef. variation	184	12.02	175.91	11.71	208.9	10.71	328.31	9.81	465.42	9.51		78.12	298.61
Skewness	4.72	−1.01	5.61	−0.96	6.31	−1.03	7.11	−1.32	7.91	−2.02		15.22	7.91

Spline predicted data represents soil profile data from the Danish Soil Profile Database, whereas point observation represents data from the Danish Soil Classification.

**Figure 2:**
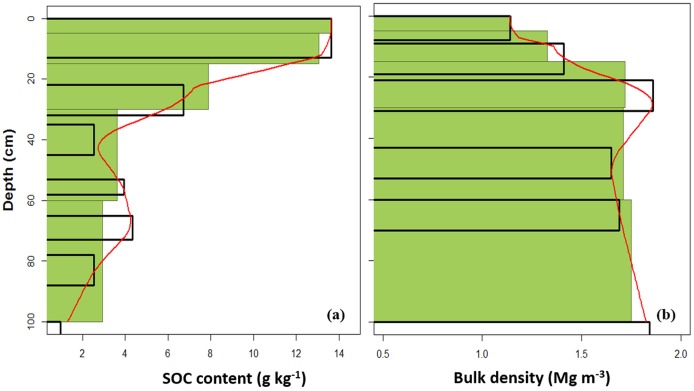
Example of a fitted spline for soil organic carbon content (a), and for bulk density (b). Horizontal bars represent measured soil organic carbon and bulk density at different soil horizons, continuous line through horizons represents a fitted spline, and horizontal olive-green bars give an weighted-average values of these properties at five standard soil depth intervals (i.e., 0−5, 5−15, 15−30, 30−60 and 60−100 cm).

Figure 2 shows a measured and spline predicted SOC and *D_b_* for a coarse sandy soil under agriculture area from the Saalian moraine soilscape in western Denmark (West Jutland). Measured SOC from different horizons decreased with depth except at 53−73 cm where it increased due to podsolization. Although a spline should pass through the mid-point of each measured horizon for this soil profile, in this case the spline slightly extrapolated the SOC value at 35−55 cm due to the selected λ at 0.1.

### Prediction rules for SOC and D_b_

Depending on the soil depth, 17 to 54 condition-based regression rules were generated while predicting SOC and *D_b_*. This paper only included one of the 54 rules produced during SOC prediction at 0−5 cm soil depth, as an example.

Rule 3: [400 cases, mean 3.1, range 2.2 to 4.5, est err 0.21]
 if
   Georegions in (1, 2, 3, 6)
   Landscape in (5, 6, 7)
   MrVBF>6.6
   Soil map in (3, 4, 6, 7)
 then

   log SOC g kg^−1^ = 1.527+0.124*MrVBF+0.0067*Elevation+0.084*SAGA wetness index–0.0011*precipitation+0.06*slope gradient.

This rule used elevation, SAGA wetness index, MrVBF, precipitation and slope gradient to predict SOC in the areas where the MrVBF index is higher than 6.6 and consisted of fine sandy soils from a Moraine landscapes from the geo-regions (e.g. Himmerland). This rule was only valid for 400 locations where the mean SOC was 3.1 [log SOC g kg^−1^] and the prediction error was about 0.2 [log SOC g kg^−1^].

### Identifying predictors

Variables were identified for SOC and *D_b_* prediction based on their relative usages in the model (Table 4). In all models, precipitation appeared to be the most dominant variable followed by altitude above channel network and SAGA wetness index to predict SOC. As an example, to predict SOC at 5−15 cm, precipitation had a usage of 98% for both rule setting and developing a linear prediction model followed by SAGA wetness index which had a relative usage of 67% and 94%. For this model, insolation and slope aspect had the lowest contribution. Geology became robust with increasing soil depth, whereas land use was important for SOC prediction of the surface layers. Geology, soil map, wetlands, land use, precipitation, MrVBF, SAGA wetness index, elevation, slope gradient, slope-length factor, and altitude above channel network were among the predictors that had a relative importance of >60%. Similarly, land use, soil map, geology, slope gradient, SAGA wetness index, MrVBF, and elevation appeared to be the most important variables for predicting *D_b_* at all soil depths.

**Table 4:** Relative usage (%) of the environmental variables to predict soil organic carbon at different soil depths in Denmark.

Environmentalvariables	Soil depth (cm)									
	0−5		5−15		15−30		30−60		60−100	
	CR+	PF+	CR	PF	CR	PF	CR	PF	CR	PF
Wetlands	72	-	55	-	10	-	-	-	-	-
Multi-resolution valleybottom flatness index	64	67	9	81	-	52	-	33	-	5
Geo-regions	62	-	65	-	17	-	73	-	10	-
Soil map	55	-	38	-	28	-	60	-	5	-
Precipitation	54	74	98	98	4	76	66	62	20	25
Landscape	53	-	26	-	15	-	48	-	5	-
Land use	45	-	60	-	5	-	-	-	-	-
Altitude abovechannel network	37	85	11	87	82	28	7	4	-	3
Elevation	36	88	31	88	50	15	32	21	-	5
Geology	27	-	23	-	30	-	62	-	100	-
SAGA wetness index	22	94	67	94	-	70	5	56	-	2
Valley depth	9	41	16	47	-	26	-	59	-	15
Slope gradient	2	78	8	93	-	54	-	63	-	10
Mid-slope position	2	50	2	52	-	54	4	15	-	2
Flow accumulation	-	30	8	35	-	26	-	-	-	-
Slope length factor	-	85	-	89	-	31	1	52	-	-
Insolation	-	34	-	16	-	-	-	17	5	-
Slope aspect	-	22	-	2	-	15	2	26	-	-

+ CR-Variable usage in setting the rule conditions; PF-Variable usage in the linear prediction function.

### Predicted maps

**Figure 3:**
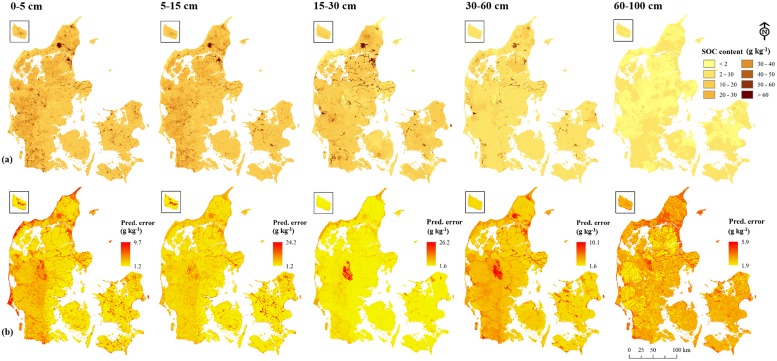
Predicted soil organic carbon content (a), and standard error maps (b) at five soil depths in Denmark.

Predicted maps of SOC content (Figure 3) and *D_b_* (map not shown) at five soil depths were produced at a resolution of 30.4×30.4 m. The highest mean SOC content was in the 0−5 cm layer (mean 20 g kg^−1^; sd 11 g kg^−1^). Predicted SOC content decreased with soil depth and at 60−100 cm, it was on average 2.2 g kg^−1^. The soils of western Denmark have relatively higher SOC content than the rest of the country. The northern part has a moderate amount of SOC with two large raised bogs with high SOC contents. Along the coastline, especially in the west, soils with lower SOC concentration were mapped.

The prediction errors were higher towards the west and along the coastline. The prediction error increased with soil depth. For the 0−5 cm layer, the mean error was 1.1 g kg^−1^ and it increased to 1.8 g kg^−1^ at 60−100 cm soil depth.

Predicted SOC content for the FAO−UNESCO soil groups is shown in Table 5. Average SOC content ranged between 11.8 to 52.6 g kg^−1^ at 0−30 cm and between 1.9 to 37.5 g kg^−1^ at 30−100 cm. The highest SOC was observed in Histosols and the lowest in Arenosols at most soil depths.

**Table 5:** Predicted soil organic carbon content (g kg^−1^) at five soil depths for each FAO-UNESCO soil groups.

FAO soil groups	Soil depth (cm)									
	0−5		5−15		15−30		30−60		60−100	
	Mean	Stdev.	Mean	Stdev.	Mean	Stdev.	Mean	Stdev.	Mean	Stdev.
Alisols	20.8	10.4	19.7	10.9	15.4	19.0	9.8	20.3	2.1	0.7
Arenosols	12.5	10.2	11.9	8.8	11.8	12.4	7.8	15.9	1.9	1.1
Cambisols	17.9	8.2	17.0	6.4	12.2	7.5	7.3	8.8	2.2	0.6
Luvisols	18.0	7.1	16.4	5.7	15.7	8.3	6.8	7.2	2.2	0.7
Podzols	21.9	10.9	21.4	14.1	16.6	25.3	9.1	17.4	2.1	1.2
Fluvisols	24.1	12.0	22.7	9.0	16.7	15.3	12.0	27.5	2.6	0.9
Gleysols	22.8	15.2	22.3	15.0	21.4	26.7	11.9	29.5	2.4	0.8
Podzoluvisols	20.8	6.3	20.8	6.6	14.7	10.2	8.7	8.3	2.0	0.7
Histosols	38.9	22.8	37.8	27.1	52.6	52.5	37.5	71.5	2.5	0.7
Unmapped areas	13.1	6.5	16.5	5.9	12.7	11.5	8.0	13.9	2.0	0.6

### Model validation

SOC prediction models performance is summarized in Table 6. The best prediction was found at 5−15 cm soil depth for both training and test data sets. The model performance at 60−100 cm was relatively poor compared to the rest of the soil depths. Negative *ME* values suggested that almost all the prediction models were negatively biased suggesting some under prediction of the mapping of SOC distribution.

**Table 6:** Model performance to predict soil organic carbon content [log SOC g kg^−1^] based on Training and Validation datasets.

Soil depth (cm)	Training data			Validation data		
	*R^2^*	*RMSE*	*ME*	*R^2^*	*RMSE*	*ME*
0−5	0.61	0.22	−0.008	0.41	0.24	−0.08
5−15	0.63	0.22	−0.006	0.42	0.24	−0.02
15−30	0.51	0.62	−0.03	0.43	0.66	−0.22
30−60	0.50	0.53	−0.05	0.29	0.56	0.02
60−100	0.28	0.47	−0.06	0.23	0.48	0.12

### SOC stocks

**Figure 4:**
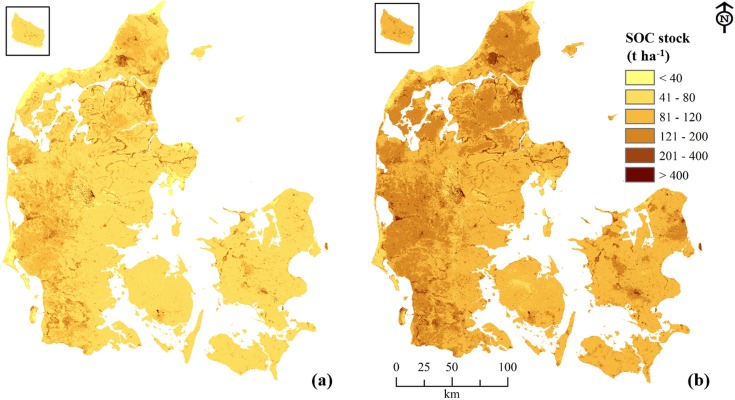
Predicted soil organic carbon stock maps at 0−30 cm (a), and 0−100 cm (b) soil depths for Denmark.

**Figure 5:**
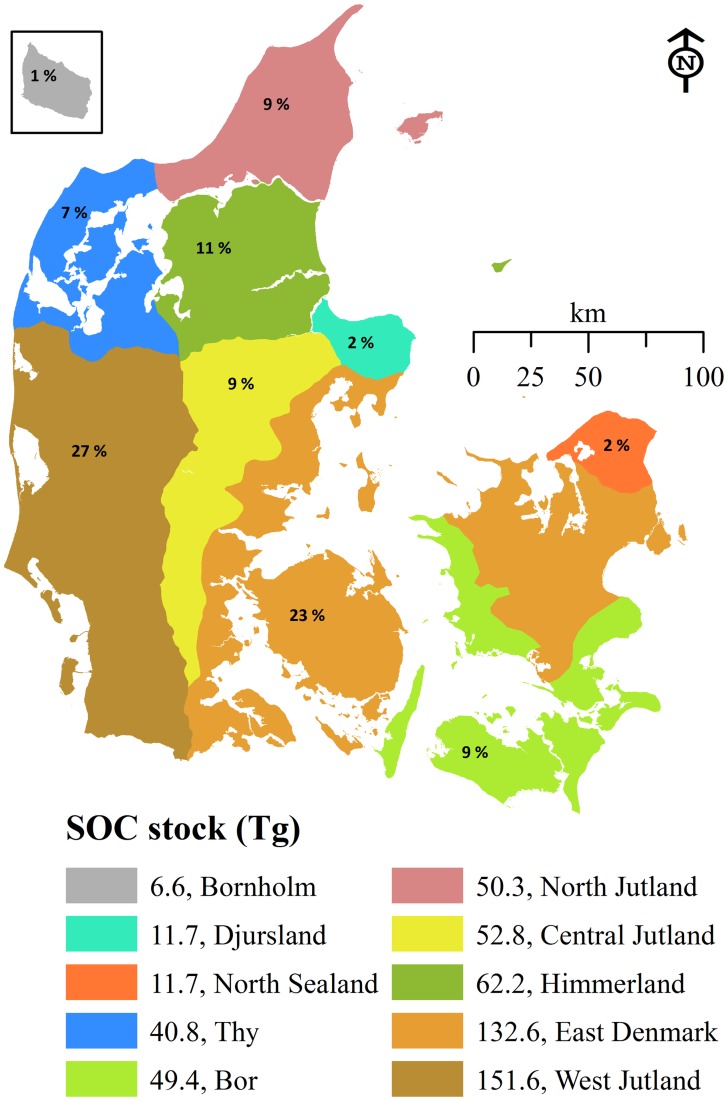
Soil organic carbon stock (1 m depth) for the geo-regions in Denmark. Percentage values represent the fraction of the total soil organic carbon content stock (570 Tg).

SOC stock maps were made for two soil depths (Figure 4). For 0−30 cm, average SOC stocks were about 72 t ha^−1^ and for the top 1 m depth, it was about 120 t SOC ha^−1^. Most of the western and northern parts of the country have more than 80 t SOC ha^−1^ in the top 30 cm whereas the average stock in the eastern part of the country was less than 80 t SOC ha^−1^. Total SOC stock was calculated for each geo-region (Figure 5) and Himmerland and West Jutland had an average stock of about 135 t SOC ha^−1^ followed by North Jutland and Thy both having a mean stock of >120 t SOC ha^−1^. The soils of West Jutland and East Denmark contain almost 50% of the total SOC stock in Denmark.

Luvisols and Podzols contain about 60% of the total SOC stock (Table 7). Other soil groups that contain significant amounts of SOC were Cambisols (6%), Gleysols (9%), and Arenosols (9%). Although Histosols had SOC stock of 176 t ha^−1^, its total content was 20 Tg SOC. For all soil groups, more than 58% of the total stock was in the top 30 cm. Unmapped areas in the soil map representing major Danish cities that may also contain a substantial amount of carbon [46].

**Table 7:** Soil organic carbon stock in the top 1 m soil depth according to FAO−UNESCO soil groups.

FAO Soil groups	Area (km^2^)	Average SOC stock (t ha^−1^)		Total stock		Relative stock
				(Tg)		(%)
		0−30 cm	0−100 cm	0−30 cm	0−100 cm	0−30 cm:0−100 cm
Alisols	921.9	71.3	118.3	7.3 (2%)	12.1 (2%)	60
Arenosols	3,585.9	60.3	105.0	29.5 (9%)	51.3 (9%)	57
Cambisols	2,910.2	64.0	109.9	20.8 (6%)	35.4 (6%)	58
Luvisols	14,499.4	62.3	107.6	100.1 (29%)	172.9 (30%)	58
Podzols	13,745.0	79.6	129.8	115.4 (34%)	189.0 (33%)	61
Fluvisols	879.6	80.2	144.5	7.7 (2%)	13.7 (2%)	56
Gleysols	3,310.0	85.3	140.5	30.3 (9%)	49.7 (9%)	61
Podzoluvisols	698.3	75.7	126.0	5.8 (2%)	9.7 (2%)	60
Histosols	1,039.6	120.8	176.1	14.0 (4%)	21.1 (4%)	69
Unmapped areas	1,320.45	63.1	109.8	9.0 (3%)	15.7 (3%)	57
Sum	42,910.6			340 (100%)	570 (100%)	

Of the total stock of 570 Tg SOC about 59% is in the upper 30 cm. Soils under agriculture have an average stock of 121 t ha^−1^ and contain about 444 Tg which is almost 78% of the total estimated stock (Table 8). Another large fraction of SOC stock is found in the soils of the forest and semi-natural areas, and these had a stock of 39 Tg in the top 30 cm and about 67 Tg up to 1 m soil depth. Wetland areas contain large amounts of SOC, and average SOC stock within 1 m soil depth was about 152 t ha^−1^ which is nearly 2% of the total stock. Almost 90% of the total SOC stock within the top 1 m soil depth is found in the soils under agriculture, forest and natural areas.

**Table 8:** Predicted soil organic carbon stock in different land use types derived for two different soil depths.

Major Land use types	Area (km^2^)	Soil depth (cm)	
		0−30	0−100
		SOC Stock in Tg	
Artificial surface (Urban, Industry, Roads, etc.)	3169.0	22.3 (6%)	38.7 (7%)
Agricultural areas	32942.3	266.4 (78%)	443.9 (78%)
Forest and semi-natural areas	5547.1	38.9 (13%)	66.7 (11%)
Wetlands	860.0	8.3 (2%)	13.5 (3%)
Other (Coastal lagoons, Estuaries, etc.)	391.9	4.1 (1%)	7.2 (1%)
Sum	42910.3	340 (100%)	570 (100%)

## Discussion

Here we have predicted the distribution of SOC contents and stocks across Denmark including an assessment of the prediction error. We have also estimated the SOC contents and stocks for different land uses and soil types. This discussion focuses on the prediction model, the importance of the variables, the uncertainty, and the SOC contents and stocks.

### Prediction model

The equal-area spline fit the discrete horizon data but it also harmonized the profile by disaggregating the horizon bulk data and generated a continuous function of SOC distribution. Several other researchers have advocated the usefulness of such splines in depth-wise mapping of SOC in different parts of the world applied from local to regional extents (e.g., [7], [8], [10]). Pegging of spline by introducing an artificial horizon on the surface benefitted our splines that restricted biased extrapolation of SOC on the surface horizon.

The spatial prediction method (i.e. rule-based regression using the *Cubist* software) was capable of exploring the relationship of SOC to its environment predictors. The prediction rules were conditioned to a given environmental settings such that each rule is valid only to that specific boundary within which SOC distribution presumably less heterogeneous compared to the areas outside where other conditions and rules prevail. For example, SOC content in forests or clayey soils might be different than the SOC from agriculture or sandy soils hence different prediction models operated in these two specific areas. Such a beneficial and advanced function has been used by Lacoste et al. [7] who found regression-rules combined with the spline depth function useful for producing a detailed pseudo-3D map of SOC content in heterogeneous agricultural landscape in France. Minasny and McBratney [42] found that regression rules provided a greater accuracy compared to partial least squares while predicting total carbon content. Several other studies have applied this tool in digital soil mapping (e.g., [35], [47], [48]).

#### Variable importance

The environmental variables used to predict SOC content showed a varying level of importance in the model. There was a large influence of precipitation, land use, soil type and some terrain parameters such as elevation, slope gradient, SAGA wetness index, and MrVBF on the spatial distribution of SOC content. The influence of topographic parameters on SOC distribution has been documented in other studies [6], [8], [9], [49]–[51]. Similarly, land use, precipitation, soil types, wetlands were found important while mapping SOC distribution [11], [14], [18], [52]–[55]. The influence of terrain parameters on SOC composition and distribution can be linked to its behavior on soil re-distribution through erosion and deposition, in the maintenance of vegetation cover and rooting depths, and in soil drainage that affects SOC decomposition as well as vegetation. In Denmark, the influence of elevation, soil types, geology, and slope gradient was also documented by Bou Kheir et al. [12] when predicting SOC in wet cultivated lands.

The categorical variables such as soil types, geo-regions, and land use were used in defining the rule conditions and continuous variables (terrain parameters) in regression functions. Some terrain parameters like elevation, SAGA wetness index, MrVBF, altitude above channel network etc. were utilized in setting rule conditions. This combined approach in defining the conditions partitioned the study area into several possible strata where SOC distribution was supposed to be more homogeneous and in each stratum several terrain parameters were again used to make sure that the within-stratum SOC variability was well captured by the model. This has made the model robust in predicting SOC content.

#### Uncertainty

Based on validation indices, the prediction models showed a higher performance (i.e., higher *R^2^* and a lower *RMSE*) in calibration data (75%) than in validation data (25%). The uncertainty of the SOC prediction increased with depth. The *R^2^* value ranged between 0.23–0.63 - the higher values for the surface layers. It suggests that our prediction was able to capture up to 63% of total SOC variability. The range of *R^2^* values was comparable to similar SOC mapping studies where internal validation was applied [24], [50], [56], [57]. Values higher than 0.7 are unusual and values <0.5 are quite common in soil attribute predictions [58]. The difference in *R^2^* value between the two datasets could probably be attributed to the different data density used in prediction. Minasny et al. [5] reviewed several previous SOC mapping studies, and reported that with increasing data density the *R^2^* of prediction was larger. Similarly, SOC maps from the upper soil depths were associated with a low prediction error compared to the maps with depth. This could be linked to the terrain parameters used as predictors because most of these parameters explain soil surface phenomena and the uncertainty increases with depth [24]. Moreover, higher data density from the surface layers (e.g., 0−5 and 5−5 cm) might have a positive influence on prediction performance.

#### SOC content and stock

SOC data from two main sources were used in this study: high-density (about 1 observation per km^2^) topsoil and subsoil samples, and from soil profiles at each 7-km grid-intersections together with the data from profiles along the pipe lines across Denmark. The grid and point data covered the entire country.

The SOC data were all from dry combustion analysis and from the period 1975−85 so we have modeled and predicted here the SOC contents and stocks for that period. Assuming a steady-state condition at that period, i.e. the carbon levels represent the equilibrium with its physical environment and landuse. Likely, SOC contents have changed since that time, thus the map can be used as a baseline to indicate spatio-temporal changes.

A higher SOC content was found in surface soils, and the lowest average SOC content of 3 g kg^−1^ was found at 60−100 cm soil depth. The western part of the country mostly consists of glacial floodplains and old Saalian moraine landscapes where soils are predominantly sandy. To increase the soil fertility in those areas, farmers has been adding for decades large amounts of manure in the form of pig slurry. This may have led to increased SOC content and gave the highest SOC stock in those areas compared to other geo-regions in Denmark as also reported in Taghizadeh-Toosi et al. [29].

Our results are in line with the previous SOC estimations in Denmark. According to Krogh et al. [26], total stock in Denmark to 1 m soil depth ranged between 563−598 Tg with 579 Tg as the average which was comparable to our prediction of 570 Tg. Similarly, SOC stock from the topsoil (0−28 cm) was about 230 Tg while our prediction showed about 266 Tg. Small differences in stocks estimates between the two studies were noticed. For example, SOC stock in agricultural areas as predicted by Krogh et al. [26] was 404 Tg, whereas the estimate in the current study is 444 Tg. This could be due to a difference in areas estimated for agricultural lands in two studies. Our prediction used CORINE data that suggested an agricultural area of nearly 32,942 km^2^, whereas this area was 28,883 km^2^ in the previous study based on AIS (Area Information System). In a separate study, Olesen [28] reported a total stock of 604 Tg from 0−60 cm soil depth calculated for an area of 34,000 km^2^ based on AIS. Unlike in our study where bulk density from the peat lands were adjusted, Olesen applied a standard bulk density of 1 Mg m^−3^ for all peats or organic soils and that might have increased the stock leading to over estimation.

Previous estimates of SOC stocks had not quantified the spatial distribution of SOC stock nor validated their prediction. Our approach seemed to be more reliable and the data generated could be useful for future SOC content and stock assessments.

A large amount of SOC was present in the soils under agriculture. Our estimation of 121 t ha^−1^ is in a comparable to the SOC stock of 111 t ha^−1^ estimated for arable lands in Scotland [59] but it was slightly higher than the arable stock in Southeast Germany [60]. Similarly, our predicted average SOC stock of 121 t ha^−1^ from the agricultural areas was lower than the findings of Taghizadeh-Toosi et al. [29]. The later study used a soil-type dependent standard *D_b_* established for 7×7 km grid and perhaps overestimated the SOC stock.

The estimation of average SOC stock in different soil groups within 0−30 cm depth was comparable to Arrouays et al. [11], but the total stock was approximately nine times less than reported for France (which is almost 13 times larger than Denmark). Contrary to our study, the soils under forest soils contained more SOC than the arable soils in France. Similar results were also reported by Chaplot et al. [13] from Laos. A slightly lower average stock in the soils under forest in our study might appear due to the inclusion of non-forested areas (semi-natural areas) in the same class and exclusion of litter layer that possibly lowered its value. Decade long intensive soil management practices such as addition of large amount of manure to the agricultural soils might have increased SOC contents and consequently the SOC stock from those areas [29]. Likewise, our estimated average stock for 1 m soil depth from different soil groups, for example, Cambisols (110 t ha^−1^), and Gleysols (140 t ha^−1^) were in a agreement with the global estimate of Batjes [22] using the same soil depth. We also observed >50% stock stored within the top 30 cm soil depth for almost all soil groups which also corresponds to the finding of Batjes [22].

Although the SOC stock in Denmark might have changed substantially over the past few decades [29], our estimation based on the available data has provided a baseline SOC. Together with the estimation of uncertainty, the maps are more reliable and could be useful in environmental research in Denmark. It could support national carbon accounting and carbon balance studies and also act as a back ground information for monitoring temporal SOC changes in Denmark.

## Conclusions

This study predicted the spatial distribution of SOC content (g kg^−1^) at five soil depths intervals (0−5, 5−15, 15−30, 30−60, and 60−100 cm) and quantified its stock (t ha^−1^) to 1 m soil depth for Denmark. DEM derivatives and soil maps were used as predictors where condition-based regression rules were applied to quantify the spatial relationship between measured SOC and *D_b_* with the predictors. The following can be concluded from this study:

- Equal area spline modeled the continuous depth function of SOC and *D_b_* data from discrete soil horizons in soil profiles from Denmark.
- The most important variables that influenced SOC distribution across Denmark were precipitation, wetlands, land use, soil types, elevation, and saga wetness index.
- Model performance was better for surface soil layers and almost all prediction models suffered from underpredictions.
- The total estimated SOC stock at 0−30 cm soil depth was about 340 Tg and that for 0−100 cm was 570 Tg.
- Almost 60% of the total SOC stock was found in Luvisols and Podzols.
- About 90% of SOC was held in soils under agriculture, forest and semi-natural vegetation. For the soils under agriculture, 60% of the SOC was found in the top 30 cm.
- West Jutland and east Denmark contained almost 50% of the total SOC stock.
- This article is an example for a national level SOC mapping based on GlobalSoilMap procedures and the methods applied can be tested and used in other part of the world.
